# The Chromatin Accessibility Landscape of Peripheral Blood Mononuclear Cells in Patients With Systemic Lupus Erythematosus at Single-Cell Resolution

**DOI:** 10.3389/fimmu.2021.641886

**Published:** 2021-05-18

**Authors:** Haiyan Yu, Xiaoping Hong, Hongwei Wu, Fengping Zheng, Zhipeng Zeng, Weier Dai, Lianghong Yin, Dongzhou Liu, Donge Tang, Yong Dai

**Affiliations:** ^1^ Department of Clinical Medical Research Center, The Second Clinical Medical College, Jinan University (Shenzhen People’s Hospital), Shenzhen, China; ^2^ The First Affiliated Hospital, Jinan University, Guangzhou, China; ^3^ Department of Clinical Medical Research Center, Guangdong Provincial Engineering Research Center of Autoimmune Disease Precision Medicine, Shenzhen Engineering Research Center of Autoimmune Disease, The Second Clinical Medical College of Jinan University (Shenzhen People’s Hospital), Shenzhen, China; ^4^ Department of Nephrology, The First Affiliated Hospital of Jinan University, Guangzhou, Guangdong, China; ^5^ College of Natural Science, University of Texas at Austin, Austin, TX, United States

**Keywords:** systemic lupus erythematosus, single-cell chromatin accessible assay, peripheral blood mononuclear cells, transcription factor, marker

## Abstract

**Objective:**

Systemic lupus erythematosus (SLE) is a complex autoimmune disease, and various immune cells are involved in the initiation, progression, and regulation of SLE. Our goal was to reveal the chromatin accessibility landscape of peripheral blood mononuclear cells (PBMCs) in SLE patients at single-cell resolution and identify the transcription factors (TFs) that may drive abnormal immune responses.

**Methods:**

The assay for transposase accessible chromatin in single-cell sequencing (scATAC-seq) method was applied to map the landscape of active regulatory DNA in immune cells from SLE patients at single-cell resolution, followed by clustering, peak annotation and motif analysis of PBMCs in SLE.

**Results:**

Peripheral blood mononuclear cells were robustly clustered based on their types without using antibodies. We identified twenty patterns of TF activation that drive abnormal immune responses in SLE patients. Then, we observed ten genes that were highly associated with SLE pathogenesis by altering T cell activity. Finally, we found 12 key TFs regulating the above six genes (*CD83*, *ELF4*, *ITPKB*, *RAB27A*, *RUNX3*, and *ZMIZ1*) that may be related to SLE disease pathogenesis and were significantly enriched in SLE patients (p <0.05, FC >2). With qPCR experiments on *CD83*, *ELF4*, *RUNX3*, and *ZMIZ1* in B cells, we observed a significant difference in the expression of genes (*ELF4*, *RUNX3*, and *ZMIZ1*), which were regulated by seven TFs (EWSR1-FLI1, MAF, MAFA, NFIB, NR2C2 (var. 2), TBX4, and TBX5). Meanwhile, the seven TFs showed highly accessible binding sites in SLE patients.

**Conclusions:**

These results confirm the importance of using single-cell sequencing to uncover the real features of immune cells in SLE patients, reveal key TFs in SLE-PBMCs, and provide foundational insights relevant for epigenetic therapy.

## Introduction

Systemic lupus erythematosus (SLE) is a chronic inflammatory autoimmune disease that affects every organ and system in the body. The loss of tolerance in the immune system results in autoantibody production, immune complex deposition, and complement activation, which lead to systemic inflammation and target tissue damage (1). Genetic factors are essential in SLE susceptibility, according to family studies and the concordance rate between twins (2). Nevertheless, gene sequences alone explain only a minority of SLE heritability. Epigenetic marks have emerged as keys to understanding a portion of the missing heritability (3), and noncoding elements within cell-type-specific genomes are vital to understanding SLE pathogenesis (4). However, little is known about the related pathogenesis.

Genetic and transcriptomic analyses have unveiled some genes and noncoding loci associated with SLE. As a result, over 80 SLE risk loci are known to influence SLE predisposition, and the majority of risk variants alter regulatory elements that govern gene expression (5). Since chromatin accessibility plays a vital role in gene regulation and genome stability, and since changes in chromatin accessibility patterns may alter the accessibility of the genome’s regulatory regions to critical proteins, chromatin accessibility patterns are emerging as an essential component of human diseases (6). Notably, the assessment of chromatin accessibility in immune cells from SLE patients lags behind that of other genome-wide measurements, such as DNA modifications or transcription (7).

The assay for transposase-accessible chromatin using sequencing (ATAC-seq) (8) technique is widely used to profile genome-wide chromatin accessibility patterns at base-pair resolution due to its simplicity and sensitivity. A plate-based ATAC-seq method for single-cell analysis (scATAC-seq) was recently developed to map open chromatin regions and identify regulatory regions (9). This method has the advantages of (i) recognizing different cell types, including subtle and rare cell subtypes, (ii) revealing cell type-specific transcription factor (TF) motifs, and (iii) allowing the exploration of cell type-specific gene regulatory networks. Using scATAC-seq to study blood-derived cells in SLE patients helps to understand the role of involved cell subsets without bias and discloses the mechanism of cell type-specific gene regulation.

Therefore, we used the scATAC-seq method to analyze the open chromatin regions of peripheral blood mononuclear cells (PBMCs) from seven SLE patients and 12 healthy controls. We identified the cell types and investigated novel and rare cell populations in SLE patients after performing cell clustering. We also parse cell-type-specific regulatory patterns and summarize TF motifs with significant differences between SLE patients and healthy controls. With Gene Ontology (GO) and Kyoto Encyclopedia of Genes and Genomes (KEGG) analyses, we explored SLE-related target genes and critical signaling pathways to provide mechanistic insights into the pathogenesis of SLE.

## Material and Methods

### Human PBMC Collection

The classification of SLE patients was based on the 2012 guidelines (10). All SLE subjects (n = 7, female, mean age 33 ± 13 years, SLE disease activity (SLEDAI) >10) and healthy controls (n = 12, male/female = 6/6, mean age 34 ± 9 years) were recruited from outpatient clinics or from among the medical staff in Shenzhen People’s Hospital (Shenzhen, China). No patient had been treated with an immune suppressant within the previous three months. The human sample studies and procedures were approved by the ethics committees of both Shenzhen People’s Hospital and Guangzhou Institutes of Biomedicine and Health (Guangzhou, China) (LL-KY-2019590) with informed written consent.

Eight milliliters of peripheral venous blood was drawn from both SLE patients and control subjects, followed by the addition of Ficoll–Hypaque solution (GE Healthcare, Switzerland) and density-gradient centrifugation. Red blood cell (RBC) lysis buffer was added to eliminate the remaining RBCs, and chilled PBS was used to wash the PBMCs. After quantification with a cell counting plate, PBMCs were stored on ice for further analysis.

### scATAC-seq Library Construction and Sequencing

As described previously (11) and as described on the manufacturer’s website https://support.10xgenomics.com/single-cell-atac, scATAC-seq libraries were generated according to the Chromium Single Cell ATAC protocol (10x GENOMICS, CG000168). In brief, the isolated nuclei were incubated with the Transposition Mix. Then, transposed nuclei, barcoded gel beads, partitioning oil, and a Master Mix were loaded on a Chromium Chip E. Next, silane magnetic beads and solid phase reversible immobilization (SPRI) beads were used. After adding a sample index (P7) and Read 2 (Read 2N) sequence, the final libraries containing the P5 and P7 primers were constructed *via* PCR with Illumina^®^ bridge amplification. Finally, Illumina^®^ sequencer compatibility, sequencing depth and run parameters, sample indices, library loading, and pooling were summarized.

### Raw scATAC-seq Data Processing

All protocols for data processing are available on the following website: https://support.10xgenomics.com/single-cell-atac/software/pipelines/latest/algorithms/overview. The main steps are as follows:

### Barcode Processing

The 16 bp barcode sequence was obtained from the “I2” index read. Each barcode sequence was checked against a ‘whitelist’ of correct barcode sequences, and the frequency of each whitelisted barcode was counted. All observed barcodes with two or fewer differences (Hamming distance ≤2) from the whitelisted sequence were scored based on the abundance of the incorrect bases’ read data and quality values. Consequently, if the probability of being the real barcode based on this model was more than 90%, the observed barcode outside the whitelist was corrected to a whitelisted barcode.

### Genome Alignment

The reference-based analysis was performed through the Cell Ranger ATAC pipeline (https://support.10xgenomics.com/single-cell-atac/software/overview/welcome). First, the adapter and primer oligo sequences were trimmed. Then, the cutadapt (12) tool was used to identify and trim the reverse complement sequence. Next, BWA-MEM (13) with default parameters was applied to align the trimmed read pairs of more than 25 bp to GRCh38.

### Duplicate Marking

Groups of read pairs across all barcodes were identified to find duplicate reads, where the 5’ ends of both R1 and R2 had identical mapping positions on the reference. Thus, the unique read pair was reported as a fragment in the file.

### Peak Calling

The combined signal from each fragment’s ends was analyzed to identify regions of the genome enriched for open chromatin. First, the number of transposition events at each base pair along the genome was counted. A smoothed profile of these events with a 401 bp moving window around each base pair and fitting a ZINBA-like mixture model was generated. The model consisted of a geometric distribution to model the zero-inflated count, a negative binomial distribution to model noise, and another negative binomial distribution to model the signal. Meanwhile, a signal threshold that determined whether a region was a peak signal (enriched for open chromatin) or noise was set based on an odds ratio of 1/5. Next, peaks within 500 bp of each other were merged to produce a position-sorted BED file.

### Cell Calling

For each barcode, the mapped high-quality fragments that passed all filters were recorded, and the number of fragments that overlapped any peak regions was determined to separate the signal from the noise. Briefly, a depth-dependent fixed count was first subtracted from all barcode counts to model whitelist contamination. Notably, this fixed count was the estimated number of fragments per barcode that originated from a different GEM, assuming a contamination rate of 0.02. A mixture model of two negative binomial distributions to capture the signal and noise was set, and barcodes that corresponded to real cells were separated from the non-cell barcodes by setting an odds ratio of 1,000. Next, a count matrix consisting of the counts of fragment ends within each peak region for each barcode was produced. The matrix was filtered to include only cell barcodes and then used in subsequent analysis.

#### Clustering and t-SNE Projection

Based on Cell Ranger ATAC, dimensionality reduction was first performed *via* latent semantic analysis (LSA) (14). Then, the data were normalized *via* the inverse-document frequency (IDF) transform. Singular value decomposition (SVD) was performed on this normalized matrix using IRLBA. Before clustering, depth normalization was carried out by scaling each barcode data point to the unit L2-norm in the lower dimensional space. Next, k-medoids clustering that produced three to six clusters and graph-based clustering and visualization *via* t-SNE were provided (15), and the data were normalized to the unit norm before performing graph-based clustering and t-SNE projection.

#### Peak Annotation

BEDtools was used to associate each peak with a gene based on the closest TSS (16). Peak-related GO enrichment analysis was performed. First, all peak-related genes were mapped to GO terms in the Gene Ontology database (http://www.geneontology.org/). Then, gene numbers were calculated for each term, and GO terms significantly enriched in peak-related genes compared to the genome background were defined by the hypergeometric test. The p-value was calculated using the following formula:

$$P=1-\;\sum_{i=0}^{m-1}\frac{\left(\begin{array}{c} M\\ i \end{array}\right)\left(\begin{array}{c} N-M\\ n-i \end{array}\right)}{\left(\begin{array}{c} N\\ n \end{array}\right)}$$

N is the number of all genes with GO annotation; n is the number of peak-related genes in N; M is the number of all genes that are annotated with the specific GO terms; and m is the number of peak-related genes in M. The calculated p-value was adjusted by the FDR, and an FDR-corrected p-value of less than 0.05 was used as the threshold. GO terms meeting this condition were significantly enriched in peak-related genes. This analysis identifies the main biological functions of related genes.

Similar to GO analysis, KEGG enrichment analysis was also performed to identify significantly enriched metabolic pathways and signal transduction pathways in peak-related genes compared to the whole-genome background. The p-value was calculated using the same equation as that in GO analysis. Notably, N is the number of all transcripts with KEGG annotation, and M is the number of transcripts annotated to specific pathways.

### TF Motif Analysis

To identify TF binding sites, the position weight matrix (PWM) of the TF motifs was obtained from the JASPAR database (17), and each peak was scanned using MOODS (https://github.com/jhkorhonen/MOODS). The threshold p-value was 1E−7, and the background nucleotide frequencies were set to be those observed in the peak regions of each GC bucket. The list of motif-peak matches was unified across these buckets to avoid GC bias in the scan. To analyze motif enrichment, the reads for a TF motif within a barcode were first counted. Then, the ratio of this number to the total read number for that barcode was calculated. Next, the value was normalized to read depth. TF motif enrichment was detected by z-scoring the distribution over barcodes of these proportion values. A modified z-score calculation using the median and the scaled median absolute deviation from the median (MAD) was performed to ensure that the analysis was robust.

### Differentially Accessible Peak Analysis

Cell Ranger ATAC was used to perform the fast asymptotic beta test used in edgeR to find differentially accessible motifs between groups of cells. For each cluster, the algorithm was run on that cluster versus all other cells, yielding a list of motifs that were differentially expressed in that cluster relative to the rest of the sample. Finally, the relative library size was computed as the total cut site count for each cell divided by the median number.

### RNA Extraction and qPCR

Three SLE patients (female, mean age 30 ± 8 years, SLEDAI >10) and three healthy controls (female, mean age 32 ± 2 years) were recruited, 8 ml of Peripheral blood was collected from each sample, and PBMCs were isolated using density-gradient centrifugation with Ficoll–Hypaque. Then B cells were isolated from PBMCs by CD19 positive selection using MACS magnetic beads (Miltenyi). The RNA was extracted from B cells using RNAiso Plus (TAKARA, 9109), chloroform, and isopropanol. Total RNA was reversed transcribed into cDNA with PrimeScript RT Master Mix (TAKARA, RR036A). The cDNA was then used for quantitative real-time PCR (RT-qPCR) analysis with SYBR Premix Ex Taq II (TAKARA, RR820A) and PCR primers. The relative expression of genes in B cells was calculated by Student’s t-test, and the differences were considered significant if the P-value was less than 0.05.

The primer sequences for genes were as follows: CD83 forward, GGTGGCTTGCTCCGAAGATG, CD83 reverse, TGACCCAGGAGACCGTGTAG; ELF4 forward, GACTGGAGTTGGACGACGTTC, ELF4 reverse, GGTGGCCTCATTGTCATCTGTC; RNUX3 forward, CGAGCATCAGCAGCCTCAG, RNUX3 reverse, TGTCCCGTAGTAGAGGTGGTAG; ZMIZ1 forward, GCAGCAGAACACCAACCAG; ZMIZ1 reverse, GTTGCCGCCTGGATTCATG; Actin forward, CATGTACGTTGCTATCCAGGC, Actin reverse, CTCCTTAATGTCACGCACGAT.

### Statistical Analysis

For data analysis, Cell Ranger ATAC software was used to perform the initial data processing and downstream analysis. As described above, Loupe Cell Browser interactive visualization software was used to generate scATAC-seq peak profiles for cell clusters. The p-values in this manuscript were calculated with Loupe Cell Browser 3.1.1 through the difference analysis feature and adjusted using the Benjamini–Hochberg correction for multiple tests.

## Results

### Cell Type Identification and Cell Type-Specific TF Motif Exploration

**Figure 1:**
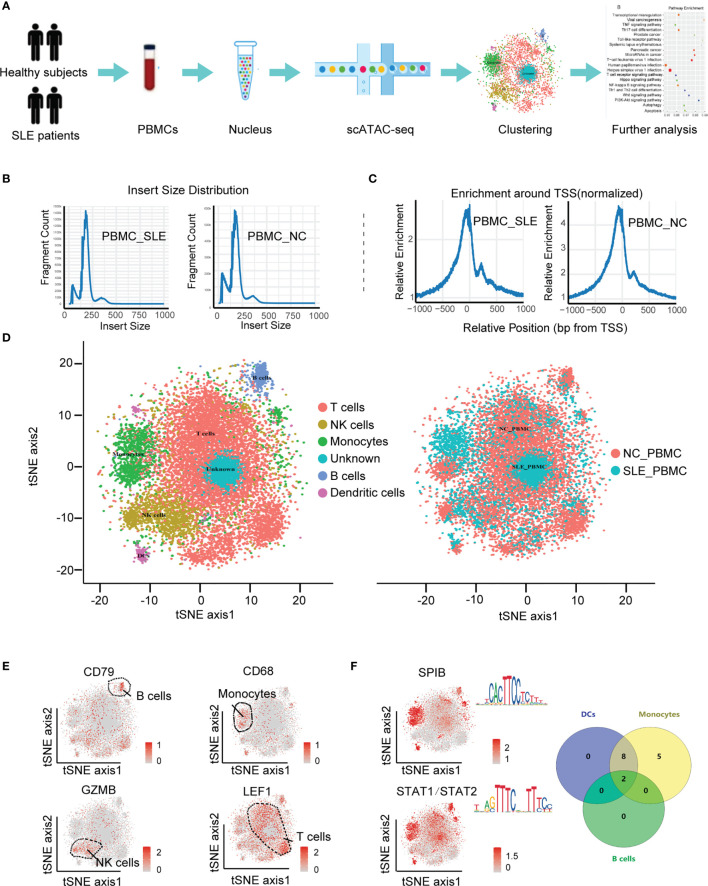
Cell-type-specific clustering of human PBMCs according to scATAC-seq. **(A)** Schematic showing the process of isolating PBMCs for scATAC-seq; **(B)** Histogram of the distribution of fragment lengths in reads from the SLE_PBMC and NC_PBMC groups; **(C)** Histograms showing enrichment of fragments at TSSs; **(D)** tSNE plot of cellular populations in the SLE_PBMC and NC_PBMC groups; **(E)** tSNE plot of canonical cell markers used to label clusters, color-coded for expression levels (gray to red); **(F)** tSNE plot of cell type-specific TF motifs (left), color-coded for expression levels (gray to red), and Venn diagram showing the distribution of 15 TF motifs with significantly differences (p <0.05) between different clusters (right). PBMCs, peripheral blood mononuclear cells; scATAC-seq, assaying transposase-accessible chromatin in single-cell sequencing; SLE_PBMC, PBMCs from patients with systemic lupus erythematosus (SLE); NC_PBMC, PBMCs from healthy controls; NK cells, natural killer cells; DCs, dendritic cells; TF, transcription factor; TSSs, transcription start sites. The p-values were calculated with Loupe Cell Browser 3.1.1 through the difference analysis feature and adjusted using the Benjamini–Hochberg correction for multiple tests.

To construct the landscape of cell type-specific open chromatin features in SLE patients, we used the scATAC-seq method to analyze PBMCs in both SLE patients (PBMC_SLE) and healthy controls (PBMC_NC) (**Figure 1A**). It is desirable to have the same sex ratio for the SLE patients and HCs (18). Since we used a fresh sample for higher data quality, our study focuses on the general landscapes of immune cells instead of cell heterogeneity in SLE patients. In addition, the results of scATAC-seq from six healthy male controls plus six healthy female controls were similar to those from seven healthy female controls. Thus, it is acceptable to further analyze PBMCs from seven SLE patients and 12 healthy controls (**Table 1**). As a result, we observed periodic peaks as nucleosome-bound fragments (**Figure 1B**) and enrichment around transcription start sites (TSSs) for both the PBMC_SLE and PBMC_NC groups (**Figure 1C**). Meanwhile, we obtained 4,993 cells from the PBMC_SLE group and 8,393 cells from the PBMC_NC group.

**Table 1:** Clinical features of patients with SLE and NC studied for scATAC-seq experiments.

Clinical characteristic (number of samples)	SLE (n = 7)	HC (n = 12)
Age (years)	33 ± 13	34 ± 9
Sex, Female/Male	7/0	6/6
Joint injury	7/7	NA
Skin lesions	3/7	NA
Hematologic abnormalities	2/7	NA
dsDNA (IU/ml)	710 ± 474	<24
ANA (AU/ml)	3,462 ± 1,824	<32
SLEDAI	>10	NA

SLE, systemic lupus erythematosus; NC, healthy controls; NA, not applicable; SLE disease activity (SLEDAI) >10.

Among the 13,386 cells, we captured a median of 5,344 unique fragments per cell. The fraction of fragments overlapping the targeted region, including TSSs, enhancer regions, promoter regions, and nucleosome-free regions, was 49.6%. The fraction of transposition events in peaks in cell barcodes was 18.3%. The total number of mapped read pairs and unique fragments in the PBMC_SLE library were 174,494,332 and 158,667,523, respectively, and in the PBMC_NC library, 193,333,851 and 165,563,843, respectively. After peak analysis, we used Cell Ranger ATAC for dimension reduction and t-SNE projection. As a result, we divided 13,386 cells into six clusters.

Based on the activity of cell marker genes, we identified and annotated five clusters (19, 20). They were T cells (cluster 1), natural killer (NK) cells (cluster 2), monocytes (cluster 3), B cells (cluster 5), and dendritic cells (DCs) (cluster 6). Interestingly, we did not observe any enriched markers in cluster 4 (**Figures 1D, E**). Since we purified PBMCs to analyze lymphocytes and all data passed quality control, cluster 4 should be an immune cell subtype. Furthermore, cluster 4 seems to be a part of cluster 1 (T cells), and our UMAP results confirmed this hypothesis. Thus, cluster 4 was regarded as a T cell subtype. Taking genes based on the enriched peaks in cluster 4 (p <0.05, |FC| >1.2) into consideration, this T cell subtype was involved in the metaphase/anaphase transition of the cell cycle (**Figure 3A**). Our results indicated that cluster 4 represented proliferative T cells (**Table 2** and **Supplementary Figure S1**).

**Table 2:** Identified markers and transcription factors in each cluster for scATAC-seq experiments.

Clusters	Cell types	Markers	Transcription factors
1	T cells	*CD3D*, *CD3G*, *CD8A*, *IL2RA*	None
2	Natural killer cells	*GZMB*, *KLRD1*, *NKG7*	None
3	Monocytes	*CD14*, *CD68*, *ITGAM*	EHF, ELF1, ELF3, ETV4, EWSR1-FLI1, GABPA, IKZF1, IRF1, KLF5, MAZ, SPIB, SPI1, STAT1::STAT2, ZKSCAN5, ZNF263,
4	Unknown (Proliferative T cells)	None	None
5	B cells	*CD79A*, *CD79B*, *MS4A1*	IRF1, STAT1::STAT2
6	Dendritic cells	*CST3*	EHF, ELF1, ELF3, EWSR1-FLI1, IKZF1, IRF1, SPIB, SPI1, STAT1::STAT2, ZKSCAN5

To explore cell type-specific TF motifs, we summarized the motifs that were significantly enriched in each cluster of PBMC_SLEs (p <0.05 and fold change (FC) >1.2). Notably, these TF motifs were not significantly different between the PBMC_SLE and PBMC_NC libraries (p >0.05). In total, we found two TF motifs in B cells, 15 TF motifs in monocytes, and 10 TF motifs in DCs (**Figure 1F**). Moreover, the 15 TF motifs in monocytes included the 10 TF motifs in DCs, and the ten motifs in DCs included the two motifs in B cells. Regarding the remaining two clusters (T cells and NK cells), there was no TF motif with an FC value of more than 1.2 (**Table 2**).

### Comparison of Open Chromatin Patterns Between PBMC_SLE and PBMC_NC

**Figure 2:**
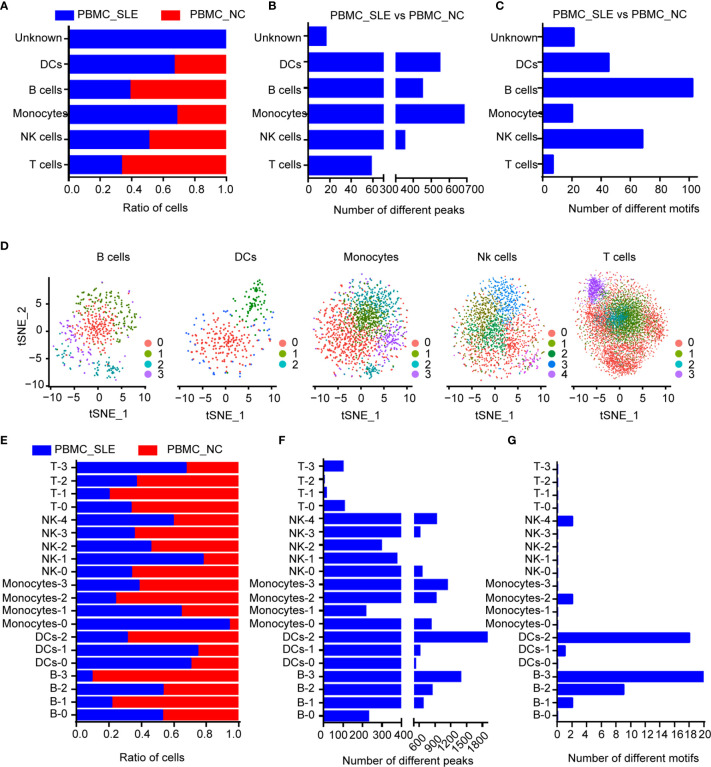
Epigenomic analysis of human PBMCs. **(A)** Cell ratios in each cell type for comparison between the SLE_PBMC and NC_PBMC libraries; **(B)** Number of different peaks in each cell type for comparison between the SLE_PBMC and NC_PBMC libraries (p <0.05); **(C)** Number of different TF motifs in each cell type for comparison between the SLE_PBMC and NC_PBMC libraries (p <0.05); **(D)** tSNE plot of B cells, DCs, monocytes, NK cells and T cells, color-coded by their associated subcluster; **(E)** Cell ratios in each subcluster for comparison between the SLE_PBMC and NC_PBMC libraries; **(F)** Number of different peaks in each subcluster for comparison between the SLE_PBMC and NC_PBMC libraries (p <0.05); **(G)** Number of different TF motifs in each subcluster for comparison between the SLE_PBMC and NC_PBMC libraries (p <0.05); PBMCs, peripheral blood mononuclear cells; SLE_PBMC, PBMCs from patients with systemic lupus erythematosus (SLE); NC_PBMC, PBMCs from healthy controls; NK cells, natural killer cells. The p-values were calculated with Loupe Cell Browser 3.1.1 through the difference analysis feature and adjusted using the Benjamini–Hochberg correction for multiple tests.

When calculating the cell ratios in both the PBMC_SLE and PBMC_NC groups, we found a significant difference in T cells and B cells (Student’s t-test, p <0.05, FDR <0.05). Notably, proliferative T cells (cluster 4) existed only in PBMCs from SLE patients (**Figure 2A**). Then, we counted the number of significantly different loci in each cluster (Student’s t-test, p <0.05, |FC| >1.2). We observed 2,092 significantly different peaks between the PBMC_SLE and PBMC_NC·libraries. In detail, there were 447 significantly different peaks in B cells, 544 in DCs, 680 in monocytes, 347 in NK cells, 58 in T cells, and 16 in the unknown group (**Figure 2B**). In addition, we calculated the number of significantly enriched motifs in each cluster (p <0.05, FC >1.2). We obtained 7, 68, 20, 102, 45, and 21 significantly enriched motifs in T cells, NK cells, monocytes, B cells, DCs, and the unknown cluster, respectively (**Figure 2C**). Our results indicated that both proliferative T cells and non-T cells were active in SLE patients.

We then reclustered T cells, NK cells, monocytes, B cells, and DCs separately for further analysis. In addition, we obtained 20 subclusters (**Figure 2D**). The cell number ratios of subcluster 0 in monocytes (Monocyte-0) and subcluster 1 in NK cells (NK-1) increased by more than three times in the PBMC_SLE group compared with the PBMC_NC group. In contrast, the cell number ratios of subcluster 1 (B-1) and subcluster 3 (B-3) in B cells, subcluster 2 in monocytes (Monocyte-2), and subcluster 1 in T cells (T-1) decreased by more than three times (**Figure 2E**). Interestingly, the number of significantly different peaks in each subcluster of NK cells, monocytes, B cells, and DCs was much larger than that in the T cell subsets (p <0.05, |FC| >1.2, 748 ± 426 *vs.* 53 ± 47) (**Figure 2F**). Similarly, the number of significantly enriched motifs in each subgroup of NK cells, monocytes, B cells, and DCs was much larger than that in the T cell subgroups (p <0.05, FC >1.2, 72 ± 46 *vs.* 7 ± 8), except for subgroup 3 in monocytes (Monocyte-3), which showed 12 significantly enriched motifs. Meanwhile, seven subclusters showed enriched motifs with a FC greater than 2 (**Figure 2G** and **Table 3**).

**Table 3:** Subclusters with enriched motifs in PBMC_SLE for scATAC-seq experiments.

Subclusters	Top 3 enriched motifs in each subcluster(p <0.05, FC >1.2)	Enriched motifs in PBMC_SLE (p <0.05, FC >2)	
		ID	Transcription factors
B-1	MA1125.1, MA0684.2, MA0025.2	MA0057.1, MA1100.2	MZF1(var.2), ASCL1
B-2	MA0500.2, MA0080.5, MA1635.1	MA0089.2, MA0501.1, MA0659.2, MA1640.1, MA0495.3, MA0846.1, MA0003.4, MA0032.2, MA0808.1	NFE2L1, MAF::NFE2, MAFG, MEIS2(var.2), MAFF, FOXC2, TFAP2A, FOXC1, TEAD3
B-3	MA0080.5, MA0687.1, MA0081.2	MA1535.1, MA0663.1, MA0649.1, MA0101.1, MA0829.2, MA0806.1, MA0807.1, MA0664.1, MA0508.3, MA1520.1, MA1464.1, MA1643.1, MA0812.1, MA0592.3, MA1527.1, MA0149.1, MA1536.1, MA1151.1, MA1581.1, MA1521.1	NR2C1, MLX, HEY2, REL, SREBF1(var.2), TBX4, TBX5, MLXIPL, PRDM1, MAF, ARNT2, NFIB, TFAP2B(var.2), ESRRA, NFIC(var.2), EWSR1-FLI1, NR2C2(var.2), RORC, ZBTB6, MAFA
DCs-1	MA0901.2, MA1104.2, MA0076.2	MA0663.1	MLX
DCs-2	MA1528.1, MA1142.1, MA0489.1	MA0046.2, MA0725.1, MA0524.2, MA0814.2, MA0153.2, MA0811.1, MA1496.1, MA0754.1, MA0694.1, MA0787.1, MA1651.1, MA0753.2, MA0652.1, MA0671.1, MA0789.1, MA0810.1, MA0037.3, MA0003.4	HNF1A, VSX1, TFAP2C, TFAP2C(var.2), HNF1B, TFAP2B, HOXA4, CUX1, ZBTB7B, POU3F2, ZFP42, ZNF740, IRF8, NFIX, POU3F4, TFAP2A(var.2), GATA3, TFAP2A
Monocytes-2	MA0748.2, MA0506.1, MA0765.2	MA0863.1, MA0682.2	MTF1, PITX1
NK-4	MA0687.1, MA0081.2, MA0080.5	MA1529.1, MA1544.1	NHLH2, OVOL1

### Functional Analysis of Significantly Changed Peaks in PBMC_SLE

**Figure 3:**
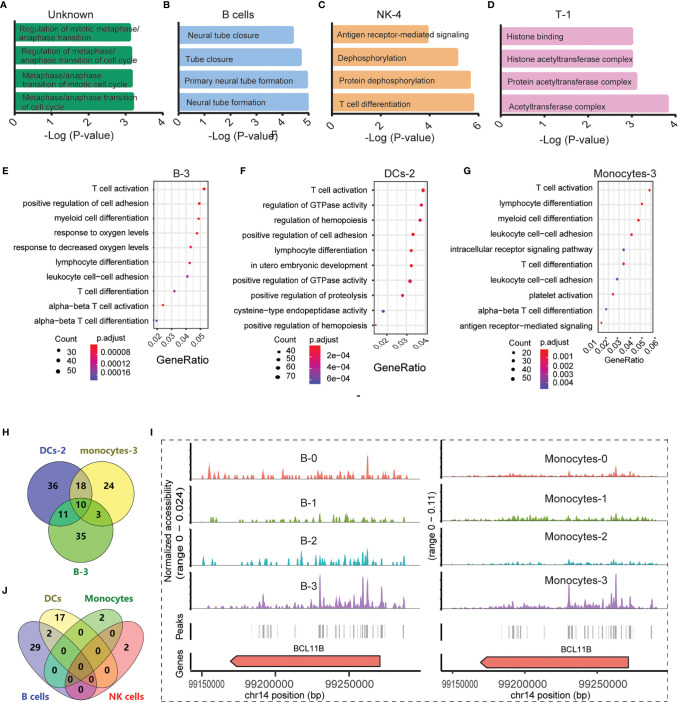
Functional analysis of significantly differential peaks between the SLE_PBMC and NC_PBMC libraries (p <0.05). **(A)** GO analysis of 16 differential genes between the SLE_PBMC and NC_PBMC libraries in the unknown group; **(B)** GO analysis of 447 differential genes between the SLE_PBMC and NC_PBMC libraries in B cells; **(C)** GO analysis of 917 differential genes between the SLE_PBMC and NC_PBMC libraries in subcluster 4 of NK cells (NK-4); **(D)** GO analysis of 11 differential genes between the SLE_PBMC and NC_PBMC libraries in subcluster 1 of T cells (T-1); **(E)** GO analysis revealing the 10 most significant pathways in subcluster 3 of B cells (B-3); **(F)** GO analysis revealing the 10 most significant pathways in subcluster 2 of DCs (DC-2); **(G)** GO analysis revealing the 10 most significant pathways in subcluster 3 of monocytes (Monocyte-3); **(H)** Venn-diagram showing distribution of genes corresponding to T cell activation in **(E–G)**; **(I)** Example locus near BCL11B with differential accessibility across B cell subclusters and monocyte subpopulations; **(J)** Venn diagram showing the distribution of 52 observed enriched TF motifs between the SLE_PBMC and NC_PBMC libraries. GO, Gene Ontology; SLE_PBMC, PBMCs from patients with systemic lupus erythematosus (SLE); NC_PBMC, PBMCs from healthy controls; NK cells, natural killer cells; DCs, dendritic cells; TF, transcription factor. The p-values were calculated with Loupe Cell Browser 3.1.1 through the difference analysis feature and adjusted using the Benjamini–Hochberg correction for multiple tests.

In B cells, DCs, monocytes, NK cells, and T cells, we chose peaks with absolute FC values greater than 1.2 for GO and KEGG analysis. The observed abnormal genes in different cells were found to be involved in various pathways. For example, B cell genes that differed between the PBMC_SLE and PBMC_NC groups were critical in neural tube formation and closure (**Figure 3B**). In contrast, the abnormal genes in DCs may play a vital role in neutrophil-mediated immunity, according to GO analysis. In monocytes and NK cells, the abnormal genes showed a high likelihood (p <0.001) of being involved in antigen processing and presentation and ubiquitin-like protein ligase binding. Meanwhile, abnormal T cell genes may be related to kinase activity and MHC class II protein complex binding.

With further GO and KEGG analysis of genes with differential expression between the PBMC_SLE and PBMC_NC groups (p <0.05, |FC| >1.2) in each subcluster of T cells, NK cells, monocytes, B cells, and DCs, we obtained more detailed information. In detail, the abnormal genes in B-0, B-1, B-2, and B-3 may be involved in axon part, the regulation of RNA splicing and apoptotic signaling pathway, transcription initiation from RNA polymerase II promoter, and T cell activation, respectively. The differentially expressed genes in DC-0 and DC-2 participate in endocytosis and T cell activation in DC subclusters, respectively. Meanwhile, the differentially expressed genes in Monocyte-0, Monocyte-1, Monocyte-2, and Monocyte-3 were active in neutrophil-mediated immunity, Toll-like receptor signaling pathways, MAPK signaling pathway, and T cell activation, respectively. In NK cell subclusters, the differentially expressed genes in NK-0, NK-1, NK-2, NK-3, and NK-4 were critical in autophagy, kinase regulator activity, negative regulation of phosphorylation, transferase activity, and negative regulation of dephosphorylation, respectively (**Figure 3C**). In T cell subclusters, the abnormal genes in T-0 and T-2 were related to histone binding and Lys63-specific deubiquitinase activity, respectively (**Figure 3D**). Notably, we did not find an available record with significantly enriched peaks in DC-1, T-0, and T-2.

Unexpectedly, in SLE patients, B-3, DC-2, and Monocyte-3 not only showed a decrease in cell number ratio but also displayed obvious abnormal signals related to T cell activation, with 59, 75, and 55 differentially expressed genes between the PBMC_SLE and PBMC_NC groups (p <0.05, |FC| >1.2), respectively (**Figures 3E–G**). As mentioned above, proliferative T cells (cluster 4) were found only in PBMCs from SLE patients. Thus, genes contributing to T cell activity may be important factors that drive T cell proliferation and lead to an abnormal immune response in SLE patients. After deduplication, there were 137 genes in B-3, DC-2, and Monocyte-3, which were involved in T cell activity (**Figure 3H**). Meanwhile, ten genes (*BCL11B*, *CCR7*, *CD83*, *ELF4*, *ITPKB*, *NCK2*, *NKAP*, *RAB27A*, *RUNX3*, *ZMIZ1*) were found in all three subgroups, which suggests that these genes should be prioritized as targets for therapy (**Figure 3H**). Further analysis indicated that *BCL11B* was highly enriched in both B-3 and Monocyte-3 compared with other B cell subclusters and monocyte subclusters, respectively (**Figure 3I**). This result indicates that *BCL11B* may be used as a marker to purify B-3 and Monocyte-3 cells from B cells and monocytes, respectively.

### Exploration of Key TFs in PBMC_SLE

**Figure 4:**
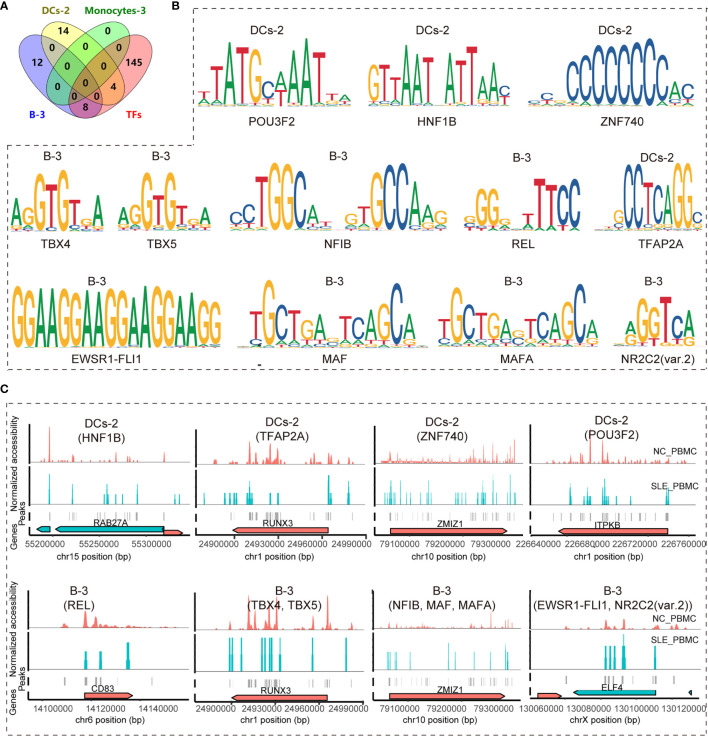
Identifying key TF regulators and their regulatory networks involved in the T cell activation pathway. **(A)** Venn diagram showing the distribution of enriched TF regulators in B-3, Monocyte-3, and DC-2 between the SLE_PBMC and NC_PBMC libraries (p <0.05, FC >2) and the 157 TF regulators involved in regulating the target genes (BCL11B, CCR7, CD83, ELF4, ITPKB, NCK2, NKAP, RAB27A, RUNX3, and ZMIZ1) to activate T cells; **(B)** Position weight matrices (PWMs) for the shared TFs in **(A)**; **(C)** Example locus near RAB27A, RUNX3, ZMIZ1, ITPKB, CD83 and ELF4 with differential accessibility between the SLE_PBMC and NC_PBMC libraries across DC-2 and B-3. SLE_PBMC, PBMCs from patients with systemic lupus erythematosus (SLE); NC_PBMC, PBMCs from healthy controls; NK cells, natural killer cells; DCs, dendritic cells. The p-values in this manuscript were calculated with Loupe Cell Browser 3.1.1 through the difference analysis feature and adjusted using the Benjamini–Hochberg correction for multiple tests.

Since we found ten genes that may be important for understanding SLE pathogenesis and further targeted therapy, the TFs regulating these genes were explored. In total, 157 TFs were found to be involved in regulating these nine genes (*BCL11B*, *CCR7*, *CD83*, *ELF4*, *ITPKB*, *NKAP*, *RAB27A*, *RUNX3*, and *ZMIZ1*) (p <0.05) according to the database, and there was no record of a TF regulating *NCK2*. We further identified the significantly enriched motifs in each subcluster of SLE patients compared to healthy controls (p <0.05, FC >1.2) (**Table 3**). In summary, there were 31 enriched motifs in B cells, including two motifs in B-1, nine motifs in B-2, and 20 motifs in B-3. In DCs, there was 1 enriched motif in DC-1 and 18 enriched motifs in DC-2. In both monocytes and NK cells, there were only two enriched motifs, one each in Monocyte-2 and NK-4. Since both B-3 and DC-1 showed the MLX motif and both B-2 and DC-2 showed the TFAP2A motif, we observed 52 enriched TF motifs in PBMC_SLE in total (**Figure 3J**). Notably, the TFs PRDM1 and IRF8, which are well-known to be associated with SLE pathogenesis, were found to be enriched in B-3 and DC-2, respectively, based on scATAC-seq analysis. When overlapping the TFs enriched in B-3, DC-2, and Monocyte-3 of SLE patients with the 157 TFs that could regulate genes to trigger T cell activity, we found 12 TFs with enriched binding sites in PBMC_SLE (FC >2), including four TFs in DC-2 (HNF1B, POU3F2, TFAP2A, and ZNF740) and eight TFs in B-3 (EWSR1-FLI1, MAF, MAFA, NFIB, NR2C2 (var. 2), REL, TBX4, and TBX5) (**Figures 4A, B** **)**. Thus, the 12 TFs may be key elements for SLE pathogenesis by regulating their target genes *CD83*, *ELF4*, *ITPKB*, *RAB27A*, *RUNX3*, and *ZMIZ1* and thereby promoting abnormal T cell activation (**Figures 4B, C** **)**.

Taking the blood cells we could obtain from both the SLE patients and healthy controls and the cell number we need for the qPCR experiments into consideration, we have only checked the expression of four genes in B cells (*CD83*, *ELF4*, *RUNX3*, *ZMIZ1*). As a result, we observed significant difference in 3 gene expression in SLE patients compared to healthy controls (*ELF4*, 0.326 ± 0.097 *vs.* 1.162 ± 0.596, p = 0.001; *RUNX3*, 0.280 ± 0.044 *vs.* 1.048 ± 0.344, p <0.001; *ZMIZ1*, 0.334 ± 0.154 *vs.* 1.119 ± 0.587, p = 0.001) (**Supplementary Figure S2**).

## Discussion and Conclusions

Since SLE is highly correlated with epigenetic modification (21), we used scATAC-seq to analyze the genome-wide chromatin accessibility of PBMC_SLE and PBMC_NC. We identified the main five cell types, namely, T cells, NK cells, monocytes, B cells, and DCs, without the use of antibodies. We also summarized the significantly enriched motifs in each cluster, which can be used as an option for cell identification. Unexpectedly, we observed a disease-specific cluster that did not express any classic markers. Through further functional analysis of the peaks enriched in PBMC_SLE, we found that this cluster was involved in the metaphase/anaphase transition of the cell cycle based on GO analysis. This result explains why we observed few markers and indicates that a state of high proliferation activity and differentiation occurred in SLE. This unknown cluster seems to be a part of cluster 1 (T cells), and our UMAP results confirmed this hypothesis. In addition, T cell activation and differentiation are typical processes in SLE patients (22). Thus, the SLE-specific cluster 4 was presumed to be proliferating T cells. Our results revealed that T cells in SLE patients experienced activation and proliferation from the perspective of open chromatin.

Based on cell reclustering, we obtained 20 accessible chromatin patterns in five main cell types. Moreover, we calculated the significantly enriched peaks and motifs in the 20 subclusters. Interestingly, the subgroups revealed detailed information different from that obtained from the five main cell types described above. For example, the abnormal genes in B cells showed functions related to neural tube formation and closure according to the GO analysis of significantly different peaks (p <0.05, |FC| >1.2). The subclusters of B cells displayed abnormal signals in the axon part (B-0), T cell differentiation, and B cell receptor signaling pathways (B-3). Similarly, we observed only seven significantly enriched motifs (p <0.05, FC >1.2) in T cells, but we found 1, 5, 0 and 20 enriched motifs (p <0.05, FC >1.2) in T-0, T-1, T-2 and T-3, respectively. This result confirms the cell heterogeneity and highlights the importance of single-cell sequencing.

B-1 showed a reduced cell number ratio, and the genes that were changed in B-1 played a role in regulating the apoptotic signaling pathway and RNA splicing, with 31 and 19 genes, respectively. In the literature, there was an increase in apoptotic debris in SLE patients, and B cells played a role in apoptotic cell clearance (23). In addition, defects in highly active B cell clearance lead to B cell tolerance (23). Our results indicate that decreased B-1 may lead to insufficient clearance of apoptotic cells and B cell tolerance loss, thereby promoting B cell activity (23). The abnormal genes in B-2 showed transcription initiation from the RNA polymerase II promoter, which was consistent with the literature reporting that the transcriptional regulation associated with the less accessible *RXRA* locus in PBMC_SLE might lead to the production of more antibodies to nuclear antigens (24). B-3 also showed a reduction in the cell number ratio and was active in the B cell and T cell receptor signaling pathways together with T helper (Th) cell differentiation (**Figure 4G**). In addition, we found that the TF motif of PRDM1 associated with B cell differentiation was significantly enriched in B-3 cells (FC >2). B cells that produce immunoglobulins are typical clinical findings in SLE patients. Meanwhile, Th cells could promote autoreactive B cells and induce immunoglobulins through cytokines and receptor binding (25). Our results suggest that B-3 is an essential subgroup in SLE pathogenesis and is highly correlated with B cell activation and antibody production. Additionally, reduced B-3 may help increase immunoglobulin production. Notably, B-0 explains why SLE often exhibits abnormal performance of the central nervous system (CNS) (26), because some B cells express genes associated with abnormal axon function.

Among the DCs, only DC-0 and DC-2 played a role in SLE pathogenesis based on functional analysis of significantly different peaks (p <0.05, |FC| >1.2). According to the literature, DCs can recognize antigens, produce chemokines, present antigens to T and B cells, absorb complex antigens and induce T and B cell immunity (21). Moreover, IRF8 was a potent repressor of BAFF, reducing autoantibodies and autoreactive B cell clones (27). Our results are consistent with the literature and show that we may find effective SLE targets by further studying DC-0 and DC-2, especially DC-2.

Similar to DCs, monocytes are antigen-presenting cells (APCs) that can present self-antigens to autoreactive cells, thereby inducing the inflammatory response of SLE patients. In addition, monocytes can secrete chemokines such as IL-1β in a TLR signal-dependent manner or produce IFNα through TLR7 signals, which indicates that monocytes and TLR-mediated pathways are essential in SLE (28). In addition, monocytes from SLE patients showed downregulation of the MAP kinase and NF-κB pathways (21). Monocyte-0 showed an increased cell number ratio and was active in neutrophil-mediated immunity. Monocyte-1 and Monocyte-3 showed abnormal Toll-like receptor signaling pathways and T cell activation, respectively. In contrast, Monocyte-2 cells showed decreases in the cell number ratio and in the effects of the MAPK signaling pathway and NF-κB signaling pathway. These results are closely related to the findings in the literature and explain the different roles of monocyte subpopulations in SLE pathology.

In NK cells, NK-0 was active in autophagy. Since both B and T cells show abnormal autophagic processes in SLE (29), NK-0 may play a role in this process. Based on the functional analysis of different peaks between PBMC_SLE and PBMC_NC, NK-1 cells showed an increased cell number ratio and abnormal signals related to kinase regulatory activity. This result was related to protein kinase activation-induced inflammation, as reported in the literature, in which NFκB-mediated and mitogen-activated kinase participate in the NK cell response (30). Binding antigens and inducing cytotoxicity through chemical modification of the substance and stimulating other immune cells (such as T cells) and secreting cytokines are typical characteristics of NK cells (31). NK-2 and NK-3 showed negatively regulated phosphorylation and transfer of the acyl group, respectively. At the same time, NK-4 showed protein dephosphorylation and T cell differentiation. This result indicates that NK-4 may be an important subgroup of NK cells in SLE due to dephosphorylation-induced cytokine secretion and the response of other immune cells.

In T cells, we did not find a functional record of T-0, nor did we observe different peaks in T-2 (p <0.05, |FC| >1.2). Since Lys63-specific deubiquitinase activity could be involved in innate immune signaling (32), T-3 may be the population that triggers this pathway. T-1 showed a decreased cell number ratio and abnormal signals related to histone binding and the histone acetyltransferase complex. This result indicates that T-1 may consist of CD4+ T cells and therefore experience a differentiating response to inflammation, as histone acetylation contributes to the expression of genes involved in regulatory T cell differentiation and function, including increased *Foxp3* stability (33).

As described above, B-3, DC-2, and Monocyte-3 cells showed obvious abnormal signals related to T cell activation. We observed T cell activation and proliferation in SLE patients. Therefore, ten genes shared in the three subgroups (*BCL11B*, *CCR7*, *CD83*, *ELF4*, *ITPKB*, *NCK2*, *NKAP*, *RAB27A*, *RUNX3*, and *ZMIZ1*) may be critical markers related to SLE disease. Based on the scATAC-seq analysis, 12 TFs with highly accessible binding sites in SLE patients were involved in regulating these genes, indicating that they may be potential targets for SLE diagnosis and treatment. Consistent with the literature, we observed that the expression of the TFs PRDM1 and IRF8 correlated with SLE pathogenesis and that these genes showed an FC value greater than 2 in PBMC_SLE. This result confirmed the accuracy of our findings. With qPCR experiments, we observe a significant difference in the expression of *ELF4*, *RUNX3*, and *ZMIZ1*. This result indicates that at least EWSR1-FLI1, MAF, MAFA, NFIB, NR2C2 (var. 2), TBX4, and TBX5 may play a key role in SLE disease pathogenesis, through regulating *ELF4*, *RUNX3*, *ZMIZ1* and mediating abnormal T cell activity.

In conclusion, we obtained 20 chromatin accessibility patterns in T cells, NK cells, monocytes, B cells, and DCs, mapping the landscape of active regulatory DNA in PBMC_SLE. Our results showed the necessity of using the single-cell sequencing method to reveal cell heterogeneity and real features. In addition, we identified ten crucial genes associated with T cell activity from B-3, DC-2, and Monocyte-3 cells and revealed their regulatory network. The 12 TFs with highly accessible binding sites regulating these genes may be critical targets for SLE diagnosis and treatments. Meanwhile, three out of four target genes in B cells have been validated to show significantly different expression between SLE patients and healthy controls. Thus, at least the seven TFs (EWSR1-FLI1, MAF, MAFA, NFIB, NR2C2 (var. 2), TBX4, and TBX5) regulating the three genes (*ELF4*, *RUNX3*, and *ZMIZ1*) may be key targets for SLE diagnosis and treatments. In the future, scRNA-seq or bulk RNA-seq coupled with cell sorting can be conducted to confirm these candidate markers, and ChIP-seq is a choice to verify the key TFs. Our results reveal candidate markers in SLE-PBMCs, demonstrate the feasibility of epigenetic therapy in patient samples, and provide foundational insights relevant to precision medicine.

## Data Availability Statement

The raw and processed data presented in the study are deposited in the Gene Expression Omnibus under accession number GSE158263.

## Ethics Statement

The studies involving human participants were reviewed and approved by the Ethics Committee of Shenzhen People’s Hospital (affiliation: Shenzhen People’s Hospital). The patients/participants provided their written informed consent to participate in this study.

## Author Contributions

HY: Investigation, writing—original draft, formal analysis, and visualization. HW: Investigation, formal analysis, and visualization. FZ and ZZ: Resources. WD: Investigation. LY and XH: Writing—review and editing. DL: Conceptualization, supervision, and funding acquisition. DT: Writing—review and editing and project administration. YD: Conceptualization, writing—review and editing, and supervision. All authors contributed to the article and approved the submitted version.

## Funding

This work was supported by the Key Research and Development Program of Guangdong Province (No. 2019B020229001), the Science and Technology Plan of Shenzhen (No. JCYJ20200109144218597), Sanming Project of Medicine in Shenzhen (No. SYJY201704 and No. SYJY201705), and Shenzhen Key Medical Discipline Construction Fund (No.SZXK011).

## Conflict of Interest

The authors declare that the research was conducted in the absence of any commercial or financial relationships that could be construed as a potential conflict of interest.

## Supplementary Material

Supplementary Figure 1 UMAP visualization of cellular populations in the SLE_PBMC and NC_PBMC groups.

Supplementary Figure 2 Relative expression of genes in B cells involved in T cell activity.
