# Supplementary figures and images for: The Chromatin Accessibility Landscape of Peripheral Blood Mononuclear Cells in Patients With Systemic Lupus Erythematosus at Single-Cell Resolution
Source: Front Immunol. 2021 May 18;12:641886. doi: 10.3389/fimmu.2021.641886 (PMC8168536; doi:10.3389/fimmu.2021.641886)

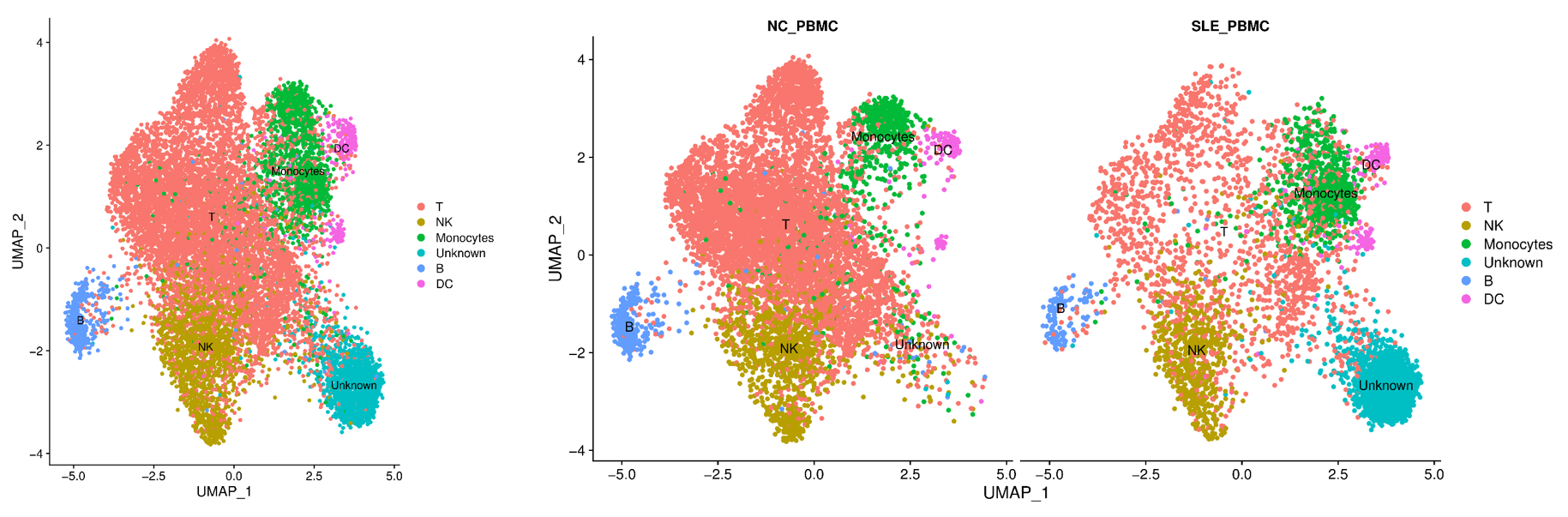

Supplement: Supplementary Figure 1 — UMAP visualization of cellular populations in the SLE_PBMC and NC_PBMC groups. [file Image_1.tif]

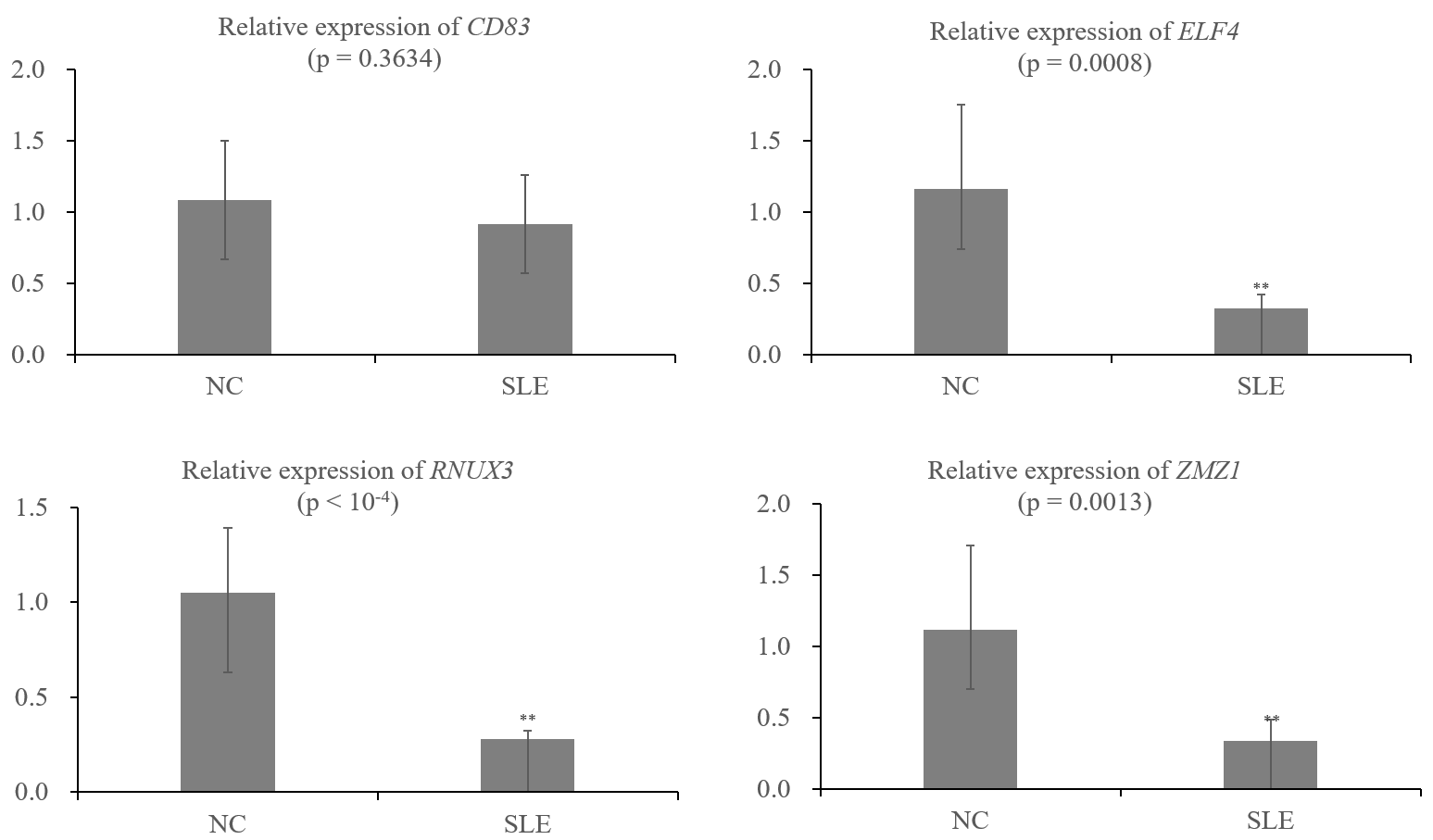

Supplement: Supplementary Figure 2 — Relative expression of genes in B cells involved in T cell activity. [file Image_2.tif]
